# Effect of Corrosion and Surface Finishing on Fatigue Behavior of Friction Stir Welded EN AW-5754 Aluminum Alloy Using Various Tool Configurations

**DOI:** 10.3390/ma13143121

**Published:** 2020-07-13

**Authors:** Abootorab Baqerzadeh Chehreh, Michael Grätzel, Jean Pierre Bergmann, Frank Walther

**Affiliations:** 1 Department of Materials Test Engineering, TU Dortmund University, 44227 Dortmund, Germany; frank.walther@tu-dortmund.de; 2 Department of Manufacturing Technology, TU Ilmenau University, 98693 Ilmenau, Germany; michael.graetzel@tu-ilmenau.de (M.G.); jeanpierre.bergmann@tu-ilmenau.de (J.P.B.)

**Keywords:** friction stir welding, aluminum alloy, load increase test, fatigue properties, corrosion, surface defects

## Abstract

In this study, fatigue behavior of surface finished and precorroded friction stir welded (FSW) specimens using various tool configurations were comparatively investigated by the load increase method. The FSW using conventional, stationary shoulder and dual-rotational configurations was carried out by a robotized tool setup on 2 mm EN AW-5754 aluminum sheets in butt joint formation. After extraction of the specimens, their weld seam and root surfaces were milled to two different depths of 200 µm and 400 µm to remove the surface and the FSW tool shoulder effects. This surface finishing process was performed to investigate the effect of the surface defects on the fatigue behavior of the FSW EN AW-5754 aluminum alloy sheets. It was found that material removal from the weld and root surfaces of the specimens, increased the fracture stresses of conventional and dual-rotational FSW from 204 to 229 MPa and 196 to 226 MPa, respectively. However, this increase could not be detected in stationary shoulder FSW. Specimens with finished surfaces, which showed superior properties, were used in salt spray and cyclic climate change test to investigate the effect of corrosion on the fatigue behavior of FSW specimens. It was shown that cyclic climate change test reduced the fatigue properties of the base material, conventional, stationary shoulder and dual-rotational FSW approximately 1%–7%. This decrease in the fatigue properties was greater in the case of the salt spray test, which was 7% to 21%.

## 1. Introduction

The joining of light metals has always been a challenge for lightweight construction industries that have high demands on the reliability and efficiency of joining technologies. One of these demands is the joining of dissimilar light metals with a low melting point such as aluminum alloys. In many cases, these materials are considered difficult to weld and do not produce suitable weld seams using conventional fusion welding techniques; consequently, these techniques cannot be used for a vast range of materials [1,2,3]. Not having the limitation of traditional welding methods, friction stir welding (FSW) presents an advantageous method for the thermal joining of various material combinations for temperatures below the melting point. This solid-state joining technology was developed at The Welding Institute (TWI) in 1991 [4], and has become an increasingly used welding process for the joining of light metals such as aluminum [5,6]. This is due to the high strength, high surface quality, low level of residual stresses and internal defects and the fine-grained structure of FSW joints. Despite the great advantages of FSW, it still requires improvement in the process force reduction, producing better surface layer conditions and avoiding unfavorable changes in the microstructure of the weld [7]. Therefore, widening the process window of FSW by removing these limiting factors is a major focus of current research [7].

**Figure 1:**
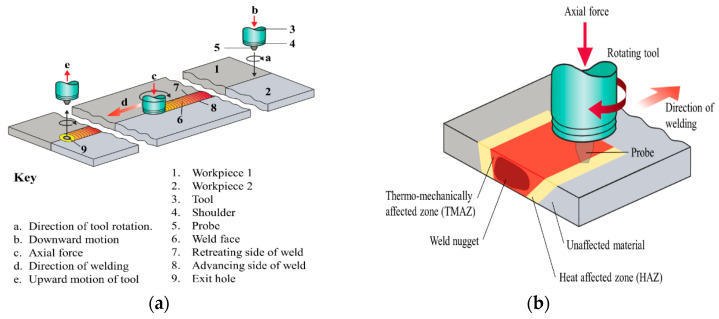
(**a**) Operating principle and components of conventional friction stir welding (FSW) and (**b**) different zones occurring during the FSW process.

The working principle of the FSW process consists of a rotating tool, which is made of a shoulder and a probe (Figure 1a). During the welding process, the probe plunges into the interface of the welding components and plastically deforms them as it moves along the designated weld seam. During the FSW process, pressure is applied by the tool shoulder to prevent material from moving out of the welding area. The plasticized materials flow around the stirring probe and in many cases create an asymmetrical material flow and temperature distribution, which degrade the fatigue properties due to unfavorable microstructure and surface properties.

**Figure 2:**
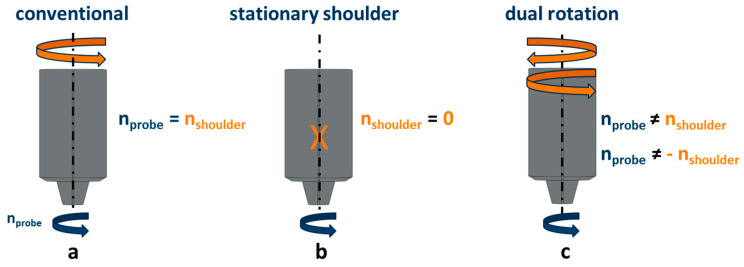
FSW with (**a**) conventional (**b**) stationary and (**c**) dual rotation tool configuration.

The welding tool in FSW has been subject to modification and optimization over the years. Figure 2 depicts varying tool configurations for the FSW process.

In the conventional design (Figure 2a), construction and handling are relatively simple and the shoulder and probe have the same rotational speed and direction (n_probe_ = n_shoulder_). This simplicity made it the most widely used design in FSW despite the rough periodic surface structures and inferior mechanical properties.

During stationary shoulder FSW, the shoulder is fixed and the probe is the only rotating part of the tool (n_shoulder_ = 0, see Figure 2b). The stationary shoulder can be used to decrease process forces due to the less material being displaced. Furthermore, a comparatively reduction of the surface roughness can be achieved due to the fact that the fixed shoulder smooths the surface across the weld seam [8]. It was found out that there exists an upward material flow in the stationary shoulder configuration FSW, which features a drain movement from the process zone through the passage between the probe and stationary shoulder up to a designated outlet ports [8]. Even though this flow enables a stable welding condition and long tool life, it leads to a certain amount of material being bled from the welding zone. However, it was shown that with a specific design of the probe, which includes threads on its shaft at the shoulder passage, the material could be forced back to the welding zone minimizing the loss of material and completely preventing the contamination of the welded part.

In the dual-rotational technique, the most recent design of FSW, the shoulder and probe can have different, independent rotational speeds and directions (n_probe_ ≠ n_shoulder_ and n_probe_ ≠ −n_shoulder,_ see Figure 2c). Process parameters and final properties of the weld such as material flow, active process force, level of plastic deformation and yield stress greatly depend on the temperature gradient during the process [9]. Therefore, parameters such as rotational speed and direction of rotation of shoulder and probe, which have a great influence on the temperature distribution within the FSW, can significantly alter the final properties of the welded samples. For this reason, the dual-rotational technique is designed to achieve a better mixture of materials and subsequent mechanical properties in FSW joints [10,11]. It was shown that dual-rotational configuration can produce the defect-free joints a wider range of welding parameters in comparison to the conventional configuration due to the unbalanced force between the upper shoulder and lower shoulder, which leads to better material flow in the FSW thickness direction [10]. This improved material flow can prevent the formation of void defect in the material [10]. However, this superiority of the dual-rotational configuration might not exist in all of the welding parameters (e.g., rotational speed of the probe, welding speed, etc.).

The general fatigue properties of materials or material compounds are represented by Wohler (S, N) curves. For the evaluation of variables that have a significant influence on fatigue properties, complete Wohler curves require a large number of specimens (and time) and only the service life (number of cycles to failure) is shown as a function of load. No information about fatigue mechanisms or the evolution and interaction of processes that lead to failure is provided.

Characteristic values for the reliable estimation of fatigue strength can be determined for various material categories and production processes using a load increase test (LIT) [12]. Compared to the conventional test methods, LIT is a single-stage testing method that provides a reliable estimation of fatigue behavior in a time- and cost-efficient manner [13]. LIT is suitable for the qualitative and quantitative evaluation of influencing variables on the fatigue behavior of FSW joints, such as plant and process parameters, surface qualities or corrosion [14]. LIT technique has been used on various materials, e.g., polymer-based materials to metallic systems, for characterization of their fatigue behavior and estimation of their fatigue strength [12,13]. It has been shown that various measurement techniques such as mechanical and electrical techniques during LIT are very well suited for the detailed characterization of the fatigue behavior of FSW aluminum composites. In LIT, the cyclic load is increased either in steps or continuously while the material behavior is being monitored using various measurement techniques until the specimen fractures.

The FSW process creates three different zones in the weld seam as shown in Figure 1b. First, the nugget zone (NZ) is formed by the shoulder and probe, whereby the workpiece components are joined by plastic deformation and dynamic recrystallization [15]. Adjacent to the NZ is the thermomechanically affected zone (TMAZ). This zone is located between the NZ and the heat-affected zone (HAZ) and undergoes only plastic deformation with no recrystallization. Finally, the HAZ, which is located between the TMAZ and the base material (BM), is only affected by the thermal cycle of the welding and undergoes microstructural changes [15]. These three regions possess different microstructures, which result in different mechanical properties [16]. It was found that different speed combinations (rotational and traverse speed) could influence the grain size at the different FSW zones, which leads to an alteration in the microhardness, mechanical properties and even the corrosion behavior. It was shown that low rotation speed and high traverse speed can increase the microhardness and corrosion resistance of AA2014 aluminum [15].

The EN AW-5754 (AlMg3) aluminum alloy was selected in the present work because of great interest in the automobile and aircraft industries as well as offshore construction applications due to its high specific strength and low density. In these applications materials are exposed to the marine environment that has a large presence of airborne salt particles and experiences a cyclic change in temperature [17,18]; hence the selection of EN AW-5754 due to its outstanding corrosion resistance to seawater and industrially polluted atmosphere [19,20,21]. Aluminum alloys are generally passive materials and pose a good corrosion resistance when exposed to neutral aqueous solutions [22]. This passivity is due to the formation and abortion of Al(OH)_3_ (aluminum hydroxide), which transforms into Al_2_O_3_·3H_2_O in neutral media and protects the material from further oxidation [22]. However, reactive materials that attack the aluminum oxide locally such as chloride [23] may remove this protective layer and expose a new fresh layer of aluminum to the corroding environment. During corrosion, aluminum atoms from the active or flawed areas dissolve on the formed barrier oxide layer [22]. The mechanism of corrosion of aluminum alloys when introduced to the neutral aqueous solutions is based on the dissolution of aluminum atoms on the naturally formed barrier film from the active sites or flawed regions [22]. It is shown that AA5754 alloy has better corrosion resistance in NaCl solution than pure aluminum due to the small fraction of iron present in aluminum [22]. It is also found that in AA 5754 aluminum alloy FSW joints TMAZ is most susceptible and HAZ is the most resistant to corrosion [20].

Many studies in the literature have investigated the fatigue behavior of friction spot welded (FSSW) EN AW-5754 aluminum alloy; however only a few focused on the fatigue properties of different FSW methods [2,24]. In addition, although several studies established the fatigue properties of aluminum alloys using the constant amplitude test (CAT), there is, to the authors’ knowledge, no studies in the literature that employed the load increase test (LIT) for the rapid assessment of the fatigue properties of FSW EN AW-5754 aluminum alloy specimens [24].

On another note, the precorrosion fatigue properties of aluminum alloys have been widely studied and established by various researchers over the past few years [25,26,27,28,29,30]. However, there are only a few studies, which investigated the precorrosion fatigue behavior of FSW EN AW-5754 aluminum alloy specimens. This is an important issue due to the fact that the weld zone is considered critical as the weakest site within the component and should receive more attention. It becomes even more critical when the weld is subjected to a corrosive environment as this has a detrimental effect on the fatigue life of entire structures by causing premature failure of components in service [31].

Another less studied aspect of FSW EN AW-5754 aluminum alloy joints are the effect of surface finishing on their fatigue properties, which can present a good solution for the components, which are subjected to extraordinary loads, repeated for millions of cycles also in extreme environmental conditions [32]. It has been shown that surface irregularities can generate local plasticity conditions, reducing crack propagation. Therefore, by surface finishing process and removal of these irregularities, fatigue resistance of the FSW components can greatly be improved [32].

The present study aimed to investigate the influence of various tool configurations and the related surface defects on the fatigue behavior of FSW welded EN AW-5754 aluminum alloy (compare Figure 2). In addition, this study set a second aim to investigate the fatigue properties of FSW EN AW-5754 aluminum alloy with and without precorroded defects by subjecting them to two different corroding environments. These investigations were carried out to extend the application of the FSW process through gaining a better understanding of influencing parameters and optimization using the efficient LIT method.

## 2. Experimental Procedure

To investigate the effect of various tool configurations (Figure 2a), the influence of surface finishing and corrosion on the fatigue properties of the friction stir welded EN AW-5754, aluminum alloy sheets with 2 mm initial thickness, 600 mm length and 170 mm width were used. The chemical composition of the material is shown in Table 1.

**Figure 3:**
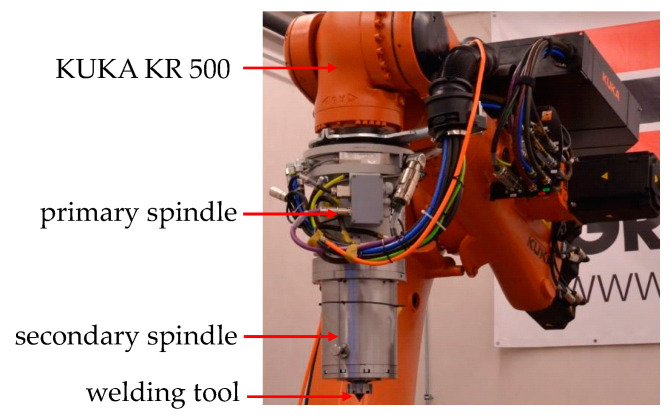
Robotized friction stir welding (FSW)-setup with double spindle construction and welding tool.

In general, all welding experiments were performed on a robotized, force controlled FSW setup from Grenzebach Maschinenbau GmbH (Asbach-Bäumenheim, Germany) using a KUKA KR 500 heavy duty robot (KUKA Aktiengesellschaft, Augsburg, Germany) with an axial force of up to 10 kN and a maximum rotational speed of up to 14000 rpm. The weld seam production was made with a double spindle construction, depicted in Figure 3.

**Figure 4:**
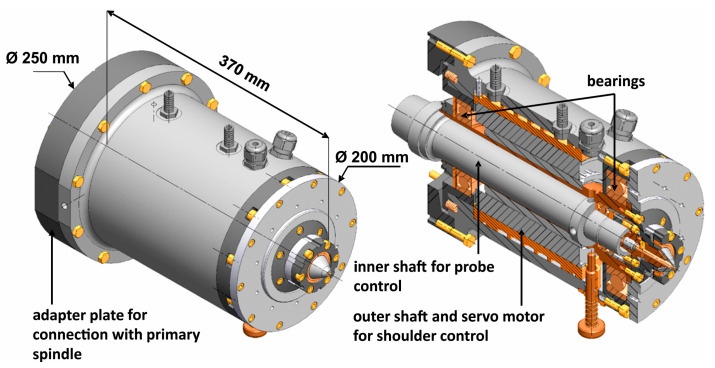
Schematic structure of the secondary spindle with inner and outer shaft to control shoulder and probe rotational speed and rotational direction.

The investigations with varying tool setups were carried out with a self-fabricated spindle construction that consisted of a primary and a secondary component [32]. The primary spindle was used to control the rotational speed and rotational direction of the probe. The secondary spindle was connected below the primary spindle and ensures a separated control of shoulder rotational speed and rotational direction. The schematic structure of the secondary spindle is depicted in Figure 4.

The secondary spindle was connected with the primary spindle due to an adapter plate, which allows individual machine integration. The continuous inner shaft was connected with the probe and hence rotational speeds of up to 8000 rpm with an equal and opposite sense of rotation could be obtained. The difference to the 14,000 rpm can be explained by component imbalances, which can lead to functional damages. In contrast to the probe, the shoulder rotational speed and direction was created by an additional servo motor, which is connected with the outer shaft. Due to the additional servomotor, it was possible to set a shoulder rotational speed of up to 4500 rpm. The welding tool had a shoulder and probe diameter of 8 and 3.3 mm, respectively. The probe was designed with a conical structure for enhanced material flow and a continuous thread on the shell surface to prevent contamination and irregularities at the joint edges; the aluminum sheets were cleaned and milled before the beginning of the welding experiments. Subsequently, the welding experiments were performed with stationary shoulder, conventional and dual-rotational tool configurations. The rotational speed of the probe was 4500 rpm for the stationary shoulder and 3500 rpm for the conventional and dual-rotational configuration for the dual-rotational configuration, the rotational direction of the probe was counterclockwise and for the shoulder clockwise. The rotational direction of the probe was kept constant in all experiments to adjust the material flow in the direction of the weld seam root. All experiments were carried out with a constant axial force of 2 kN and a welding speed of 200 mm/min.

**Figure 5:**
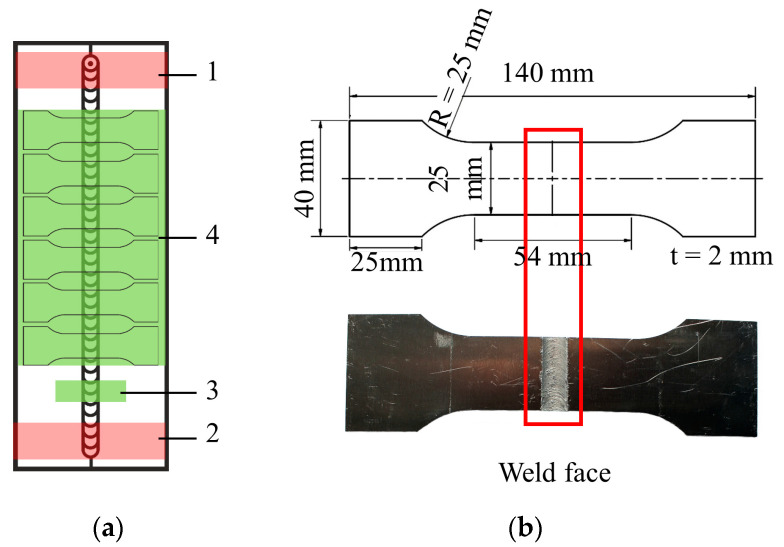
(**a**) Position of the milled specimens from the friction stir welded (FSW) aluminum sheets and (**b**) specimen geometry.

The production of the welds was repeated three times. After welding, the specimens were prepared using a milling procedure according to DIN 50,125 (Figure 5a). The two red zones 1 and 2 in Figure 5a show the first 50 mm of the FSW, which were discarded. Zones 3 and 4 were used for visual inspection of the weld and specimen production, respectively. The geometry of the specimens produced can be seen in Figure 5b.

**Figure 6:**
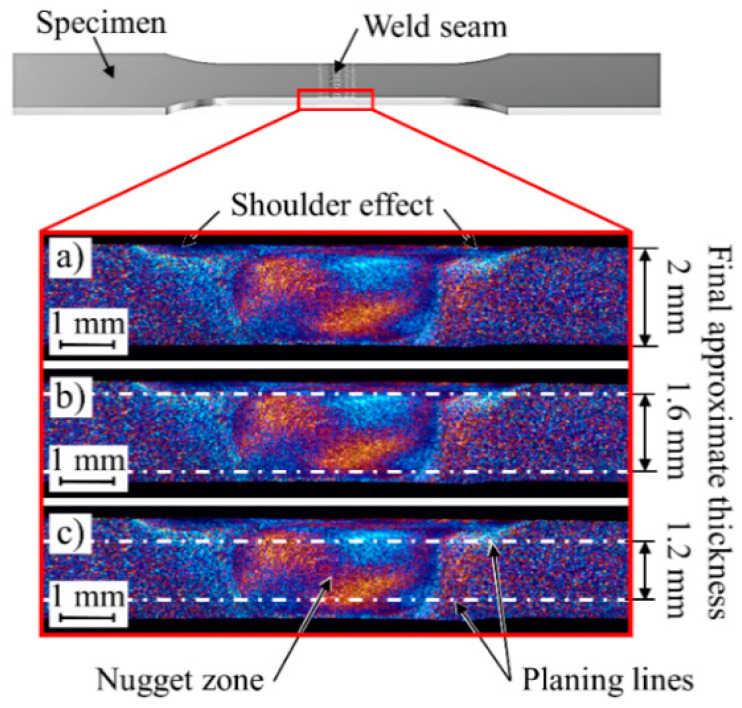
Approximate thickness of (**a**) as-welded, (**b**) 200 µm and (**c**) 400 µm finished specimens.

To avoid the influence of surface defects and to isolate the effect of the FSW microstructure on the fatigue behavior of the FSW aluminum alloy specimens, a surface finishing process was performed on both sides of the specimens (Figure 6). The milling height was determined by measurements in metallographic cross sections. Thus, material removal to a depth of (a) 200 µm (Figure 6b) to remove the surface defects and (b) 400 µm (Figure 6c) to remove the shoulder effect was carried out to investigate differences in the mechanical properties compared to the as-welded specimens. For the surface finishing process, the specimens were clamped from the shoulders and milled for a length of 80 mm in the middle of the specimens. The specimens were measured for dimensional accuracy and polished after milling to minimize the effect of the milling on the surface and edges of the specimens.

In addition to the surface finishing, the effects of a corrosive environment on the fatigue properties of the FSW aluminum specimens were also investigated. Three finished specimens (200 µm) from base material, conventional, stationary shoulder and dual-rotational FSW were subjected to a salt spray test (DIN EN ISO 9227) and a cyclic climate change test (VDA 621-415). The purpose of the cycling corrosion tests was to investigate the corrosion in accelerated laboratory tests that generate results of a corrosion effect that correlates well with those seen in vehicles or airplanes. The test particularly simulates subsurface corrosion, edge corrosion and side corrosion that emanate from damage of the surface area.

The salt spray test was performed for 1000 h using the Liebisch corrosion chamber of SK-400. The concentration of sodium chloride and the average collection rate for a horizontal collecting area of 80 cm^2^ in the test chamber were 50 g/L and 1.5 mL/h, respectively. The cyclic climate change test was performed for 5 cycles for the duration of 840 h using Liebisch corrosion chamber of SKB 400A-SC. The detail of the cycles is shown in Table 2.

**Figure 7:**
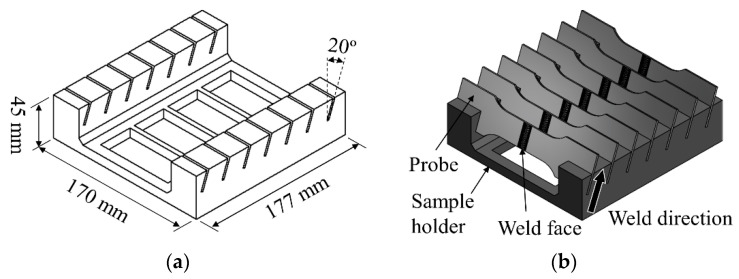
(**a**) Specimen holder geometry and (**b**) configuration of the specimens during the corrosion tests.

The specimen holders for the corrosion tests were produced in-house from polylactide with the dimensions shown in Figure 7a. To keep the effects of the corrosive environments on all the specimens the same and to ensure the reliability of the results produced, all the specimens were cleaned and placed in the same orientation with both the weld face and the direction of the weld facing upwards (Figure 7b). This allowed the specimens to be directly exposed by the salt spray in the corrosion tests. The specimens were positioned in the salt spray cabinet under the atomizer so they were not in the direct line of travel of the salt spray.

To investigate the fatigue behavior of the FSW EN AW-5754 specimens, cyclic loading tests with continuous LIT were performed. Fatigue tests were performed using an Instron 8801 servohydraulic testing system with a load cell of 100 kN.

LIT was started at a maximum stress level of σ_max,start_ = 20 MPa, which was far below the fatigue limit. Afterwards, the stress level was continuously increased from this damage-free stress level at a rate of dσ_a_/dt = 10 MPa/10^4^ with a testing frequency of 10 Hz and under the full tensional stress ratio of R = 0.1 (σ_min_ = 10% σ_max_) until specimen fracture. During the tests, an extensometer with a gauge length of 12.5 mm and a strain measurement range of ±40% was used to measure the total strain amplitude ε_a,t_ of the specimens.

**Figure 8:**
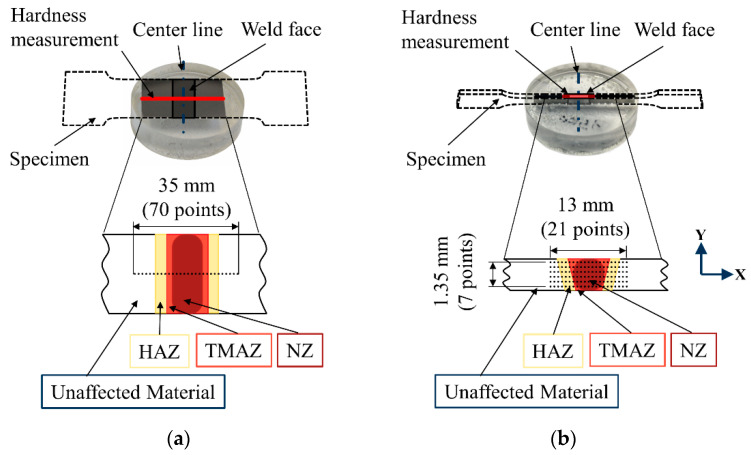
Configuration of the hardness measurement on the (**a**) surface and (**b**) the cross-section of the friction stir welded (FSW) specimens.

To understand the effect of the welding process on the hardness of the aluminum alloys, Vickers hardness measurements were performed using the microhardness measurement system of Shimadzu HMV-G-FA-D. Microhardness measurements with a force of 1.961 N (HV0.2) were performed on the polished weld surfaces to investigate the hardness variation on the weld surface of the specimen (Figure 8). In addition, microhardness mappings with a force of 98.07 mN (HV0.01) were performed on the side of the specimen to gain knowledge regarding the core of the FSW (Figure 8a). Figure 8b shows the pattern of the hardness mapping from the cross-section of the specimens. The distancing between the hardness indentations and the specimen edge was set according to the standard DIN EN ISO 6507-1.

In this study, LIT was used to assess the influence of the corrosion and surface finishing process on mechanical properties of friction stir welded EN AW-5754 aluminum sheets using different FSW tool configurations. After testing, the fracture surfaces were analyzed using a scanning electron microscope (SEM, Tescan Mira 3 XMU, TESCAN GmbH, Dortmund, Germany) with a beam acceleration voltage of 30 kV and a working distance of 25 mm to investigate the fracture mechanism. In addition, energy dispersive X-ray spectroscopy (EDX) was carried out for a duration of at least 10 h using an additional EDS-detector (EDAX Octane Pro) assessing Kα1- and Lα1-radiation.

## 3. Results and Discussion

### 3.1. Surface Finishing Process

#### 3.1.1. Fatigue Properties

**Figure 9:**
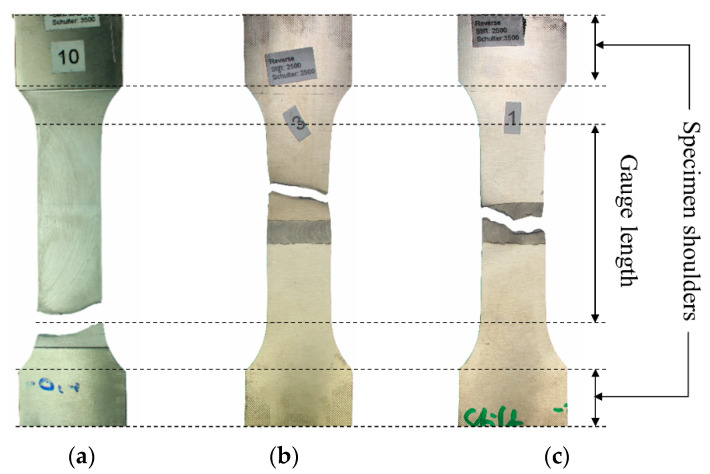
Examples of fractures (**a**) near the specimen shoulder, (**b**) in the base material and (**c**) in the middle.

**Figure 10:**
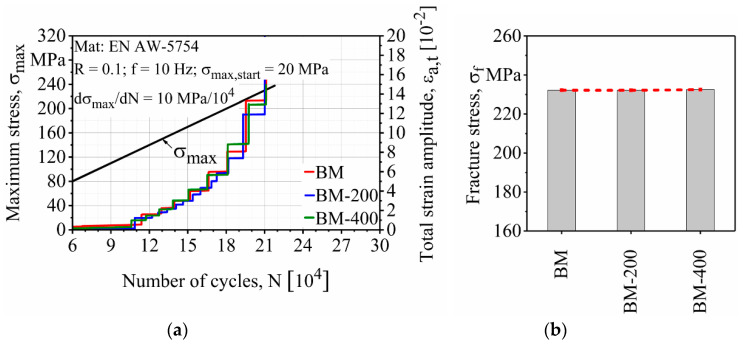
Effect of the surface finishing process on the (**a**) load increase test and (**b**) fracture stresses of EN AW-5754 base material.

**Figure 11:**
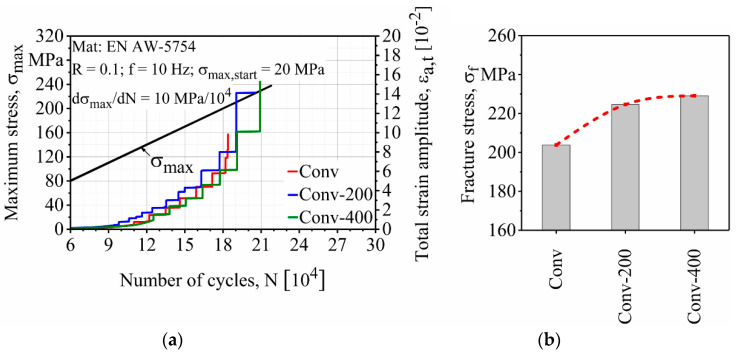
Effect of the surface finishing process on the (**a**) load increase test and (**b**) fracture stresses of conventional friction stir welded (FSW) specimens.

**Figure 12:**
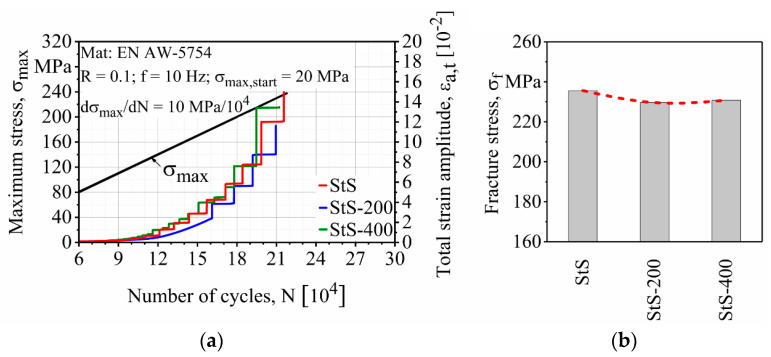
Effect of the surface finishing process on the (**a**) load increase test and (**b**) fracture stresses of stationary shoulder friction stir welded (FSW) specimens.

**Figure 13:**
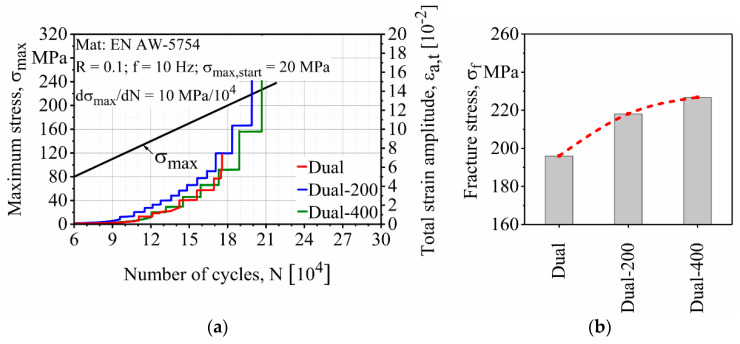
Effect of the surface finishing process on the (**a**) load increase test and (**b**) fracture stresses of dual-rotational friction stir welded (FSW) specimens.

The fractured specimens can be divided into three categories. First, the specimens that fractured near their shoulder section (Figure 9a). For the surface finishing process to take place, the specimens had to be clamped from the shoulders. Due to the limitation in the working space of the milling machine, the surface finishing process was performed in the transition zone of the shoulder and gauge section. This probably caused stress to be concentrated in a zone that resulted in the failure of the first group. Second, the specimens that failed in the base material, which was not affected by FSW (Figure 9b). Despite the fact that the specimens were all polished after the surface finishing, several specimens failed from the edges in the zone not affected by FSW. The final group is specimens that fractured from the middle or near middle section close to the welding zone; these comprise the majority of the fractured specimens (Figure 9c). The results shown in this section all came from the last group and the results of the first two groups were discarded. For some of the welding configurations that achieved more than one successful fatigue fracture (final group), the results were averaged. The effect of the surface finishing process on fatigue properties of the base material (BM), conventional (Conv), stationary shoulder (StS) and dual-rotational (Dual) FSW can be seen in the LIT results in Figure 10, Figure 11, Figure 12 and Figure 13, respectively.

LIT results show a stepwise material response that was due to the cyclic creep phenomenon associated with the Portevin–Le Chatelier (PLC) effect also known as dynamic strain aging. This phenomenon is fully explained for Al alloys in the literature [33].

As can be seen in Figure 10, the surface finishing process had almost no effect on the fatigue properties of the base material. This was to be expected due to the absence of FSW and zones locally affected by FSW. Hence material properties such as surface roughness and microstructures remained approximately the same. The only difference was the thickness of each specimen, which was considered to adapt to LIT to keep the stress level and increase the rate constant. All three categories of the base material showed almost the same response in the rate of damage evolution and fracture stress. The fracture stress of the BM, BM-200 (200 µm surface finishing depth) and BM-400 (400 µm surface finishing depth) were all 232 MPa.

The surface finishing process had a positive effect on the fatigue properties of the conventional FSW specimens. Figure 11 shows that increasing material removal depth on the surfaces of the conventional FSW specimens increased their fatigue strength. It can be seen from the results that Conv, Conv-200 and Conv-400 fractured at 204, 225 and 229 MPa maximum stress, respectively. According to Figure 11b, there is a significant increase of up to 10% in the fracture stress after surface finishing of the conventional FSW. This increase was reduced in the next surface finishing step (Conv-400), which showed a 12% increase in the fracture stress compared to the as-built state (Conv).

Figure 12 depicts the LIT result for the stationary shoulder FSW. The fracture of the StS, StS-200 and StS-400 occurred at 236, 230 and 231 MPa maximum stress, respectively. It shows that the stationary shoulder FSW in its unprocessed state possessed even higher fatigue properties than the base material (σ_f_ = 232 MPa). However, as can be seen in Figure 12b, the effect of the surface finishing process on the stationary shoulder FSW is not quite clear since no trend could be detected.

As it can be seen in Figure 13, the surface finishing process improved the fatigue properties of the dual-rotational FSW specimens as well. The results show that the surface layer removed from the dual-rotational FSW specimens increased their fatigue strength. The fracture of Dual, Dual-200 and Dual-400 occurred at 196, 218 and 226 MPa maximum stress. Figure 13b shows that Dual-200 and Dual-400 obtained an 11% and 15% increase respectively in the fracture stress compared to the as-welded state.

**Figure 14:**
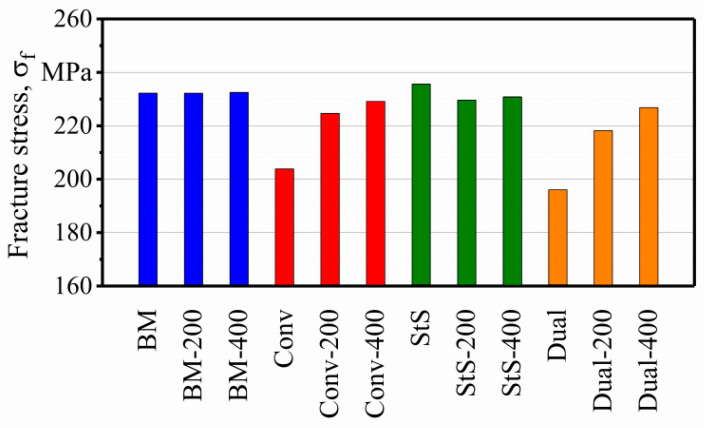
Summary of the effect of the surface finishing process on the base and friction stir welded (FSW) EN AW-5754 specimens.

A summary of the fracture stresses of all the finished specimens is plotted in Figure 14. As can be seen, even though the fatigue properties of the stationary shoulder FSW decreased after the surface finishing process, it still possessed the highest fatigue properties of all the finished and none-finished FSW specimens.

The existence of residual stresses in FSW aluminum specimens and their significant influence on fatigue properties has been reported in several studies [34,35,36,37]. It has been shown that tensile and compressive residual stresses are partially induced on the surface of conventional FSW specimens [35,37]. In the case of the stationary shoulder setup, the thermal field in the welding component was considerably narrower due to the lower heat generated by the tool and this led to a smaller zone of compressive plastic relaxation and a narrower tensile residual stress region behind the FSW tool [35]. In addition, the force applied by the stationary shoulder can also to some extent induce compressive residual stresses on the surface of the specimens. Therefore, the compressive residual stresses induced during the stationary shoulder FSW can also be a contributing factor in their superior fatigue properties. Furthermore, the slight decrease in the fatigue properties of the stationary shoulder FSW after the surface finishing process might also be due to fact that beneficial compressive residual stresses are released that make it easier for the specimen to fracture.

#### 3.1.2. Hardness Measurements

**Figure 15:**
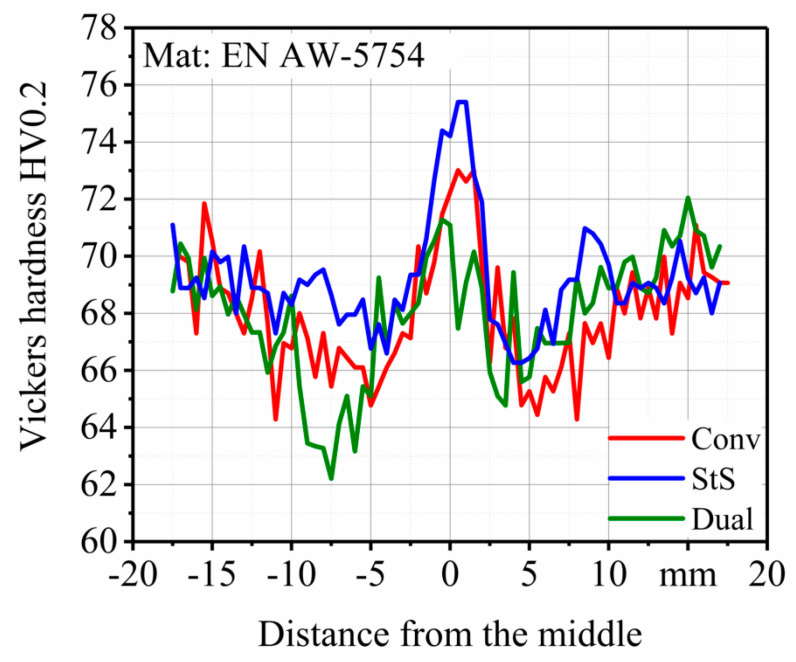
Microhardness profile at the surface of the friction stir welded (FSW) specimens.

The effect of different tool configurations on the hardness of FSW joints is shown in Figure 15. As depicted in Figure 8, measurements were performed three times across the BM, HAZ, TMAZ and NZ on the surface of the welded specimens in the transverse cross-section of the welds. It can be seen that the hardness profiles for all of the tool configurations were similar and shaped like a ‘W’, which is usual for FSW joints of aluminum alloys [37,38,39,40]. Consequently, for all of the tool configurations the maximum hardness was reached at the NZ. This is due to plastic deformation and high temperatures that lead to dynamic recrystallization and the production of fine grain structures [40]. In the case of the dual-rotational weld, the microhardness at the NZ was at the same level as the BM (70 ± 1.3 HV0.2). In the conventional and stationary shoulder configuration, microhardness at the NZ reached higher values of 72 ± 0.7 HV0.2 and 75 ± 0.4 HV0.2, respectively. After the NZ, the microhardness decreased in the TMAZ due to coarsening of the microstructure [41]. The lowest microhardness was observed in the HAZ (conventional: 66 ± 1.3 HV0.2, stationary shoulder: 68 ± 1.2 HV0.2 and dual-rotational: 65 ± 2.2 HV0.2), which was even lower than the BM. This means that heat generation during the welding process softened the HAZ due to grain coarsening. According to the results obtained in this study, microhardness decreased with an increase of grain size in compliance with the Hall–Petch relationship [37]. In the stationary shoulder configuration, the heat input to the NZ was reduced which created a smaller grain size and subsequently a higher hardness in comparison to the other tool configurations.

It can also be gathered from Figure 15 that the hardness profile depends on the tool configuration. In the conventional and stationary shoulder welds, the microhardness profiles follow a typical W-shaped path with three peaks, one at the NZ and two at the BM on both the advancing and retreating side. However, for the dual-rotational weld on the left side of the plot, there was a steep decrease in the microhardness due to the maximized heat input, which was the result of the specific rotational direction of this tool configuration.

**Figure 16:**
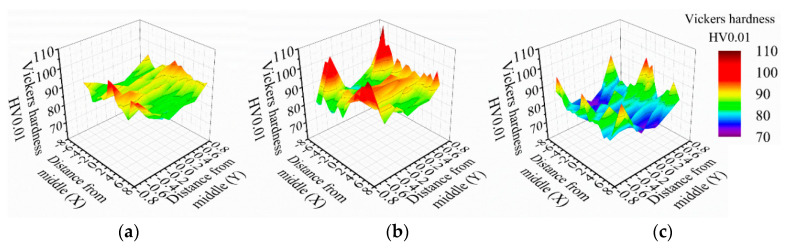
Microhardness mapping profile at the cross-section of the (**a**) conventional, (**b**) stationary shoulder and (**c**) dual-rotational friction stir welded (FSW) specimens.

The results of the microhardness mappings at the thickness of the specimens show a similar distribution pattern to the conventional (Figure 16a), stationary shoulder (Figure 16b) and dual-rotational (Figure 16c) welds. The microhardness maps show a W-shaped profile on the upper and lower sections of the welds with higher microhardness values at the edges. However, the microhardness profiles show a much smoother path in the middle of the thickness of the specimen, which suggests a more homogeneous heat input and material mixture. Conventional FSW shows the most homogeneous microhardness distribution with the lowest variation in the hardness values and the average of 89 HV0.01. The dual-rotational configuration has the highest variation in its profile with a high number of peaks at the upper and lower edges of the microhardness map. These do not indicate a homogeneous material flow and temperature gradient. The dual-rotational configuration also has the lowest average microhardness, 81 HV0.01. Stationary shoulder FSW has the highest average microhardness (over the NZ, TMAZ and HAZ), 90 HV0.01, and falls between the conventional and dual-rotational FSW in terms of homogeneous variation of the microhardness.

Another reason for the lower fatigue properties of the finished stationary shoulder FSW might lie in the existence of hard phases near the surface. Since stationary shoulder FSW does not suffer from surface defects as much as the other FSW configurations, by removing these phases, one of the obvious advantages of this FSW tool configuration is lost during the surface finishing process and a slight decrease in the fatigue properties might occur.

### 3.2. Corrosion Tests

#### 3.2.1. Corrosion Characteristics and Morphology

As part of the protocol for measuring weight loss due to corrosion, the specimens were washed, ultrasonically cleaned in ethanol for 10 min and then rinsed again at the room temperature before and after the corrosion tests. Afterwards, all of the specimens were weighed; the results are shown as a total average in Table 3.

**Figure 17:**
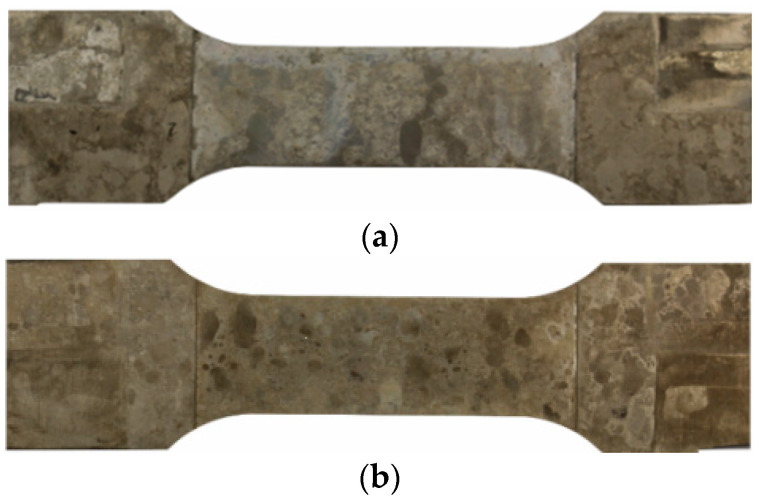
Visual inspection of EN AW-5754 specimens after (**a**) salt spray test and (**b**) cyclic climate change tests.

As can be seen from Figure 17, the surfaces of the specimens were covered by a layer of corrosion products (yellowish-brown cast on the specimens from the cyclic climate change test and slight grey-brown cast in the specimens from the salt spray test).

**Figure 18:**
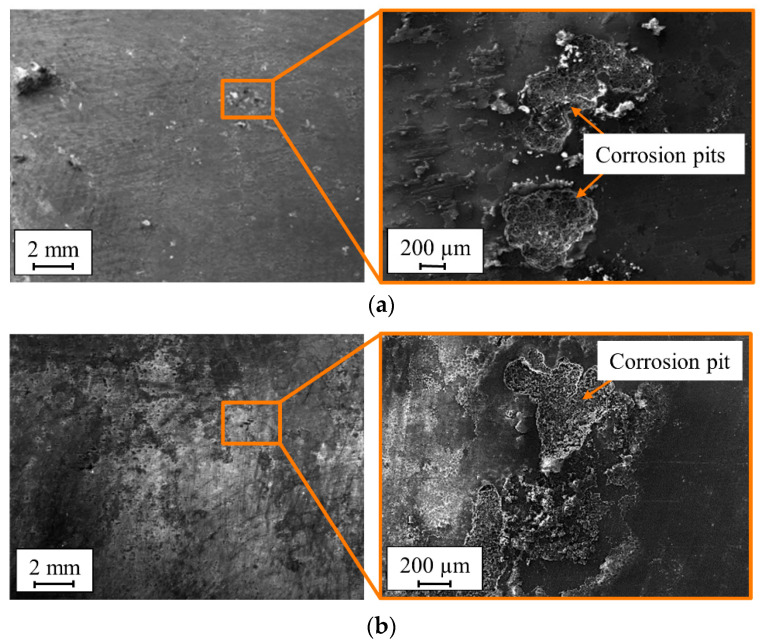
SEM images of EN AW-5754 specimens after (**a**) the salt spray test and (**b**) cyclic climate change tests.

After the visual inspection, the specimens were characterized by SEM (Figure 18). SEM observations showed multiple pits on the surface of the precorroded specimens. Cyclic climate change and the salt spray tests resulted in corrosion pits with a maximum diameter of 0.9 and 1.3 mm, respectively. These pits are the weakest spots in the protective oxide layer at which localized attacks spread spontaneously on the aluminum specimen surface. According to the literature, these spots are probably places in the material matrix of the aluminum specimen that contain iron-rich particles (e.g., Al_6_Fe, Al_3_Fe and Al_12_Fe_3_Si_2_). These intermetallic impurities, which are covered by an oxide layer process different chemical composition from the main matrix. Therefore, it is believed that they act as cathodes in aluminum matrix and when they are exposed to the salt spray (which contains chloride solution) it creates a galvanic cell that favors the pitting corrosion in the surrounding of the aluminum matrix [22]. This phenomenon has been observed in several other studies [22,23]. It can be seen that corrosion of the aluminum solid solution around the precipitates created a white corrosion product that covered each pit. Both visual and SEM observations showed that the corrosion was distributed quite homogenously on the exposed section of the specimens.

#### 3.2.2. Fatigue Properties

**Figure 19:**
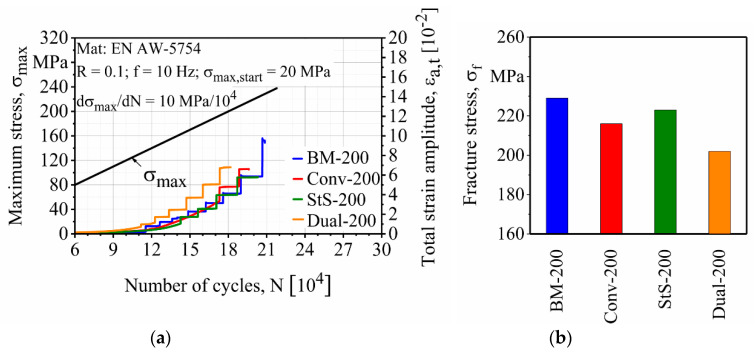
Effect of the cyclic climate change test on the (**a**) load increase test and (**b**) fracture stresses of base and friction stir welded (FSW) EN AW-5754 specimens.

**Figure 20:**
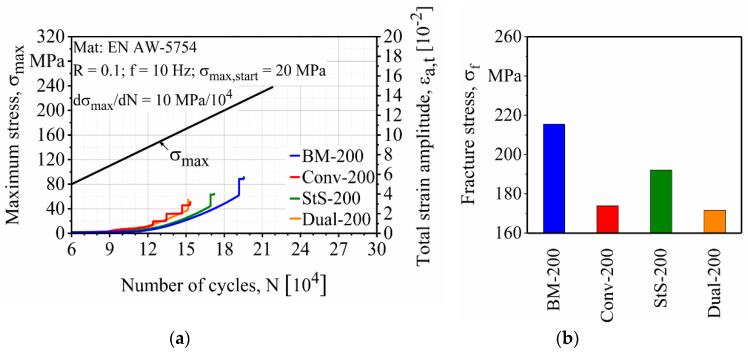
Effect of the salt spray test on the (**a**) load increase test and (**b**) fracture stresses of base and friction stir welded (FSW) EN AW-5754 specimens.

Figure 19 and Figure 20 show the fatigue properties of the precorroded specimens. It can be seen that the salt spray test had a more significant effect on the reduction of the fatigue properties of the FSW EN AW-5754 specimens than the cyclic climate change test. This was expected since higher weight loss and bigger corrosion pits were created by the salt spray test.

For both precorroded specimens, base materials showed the best fatigue properties with σ_f_ = 229 and 215 MPa after the cyclic climate change and salt spray tests, respectively. Fatigue properties of the precorroded FSW specimens show the same trend as the finished and uncorroded specimens. Stationary shoulder shows the best fatigue properties among the other FSW specimens with σ_f_ = 223 and 192 MPa after the cyclic climate change and the salt spray tests, respectively. It is followed by the conventional FSW with σ_f_ = 216 and 174 MPa and dual-rotational FSW with σ_f_ = 202 and 172 MPa after cyclic climate change and salt spray tests, respectively.

**Figure 21:**
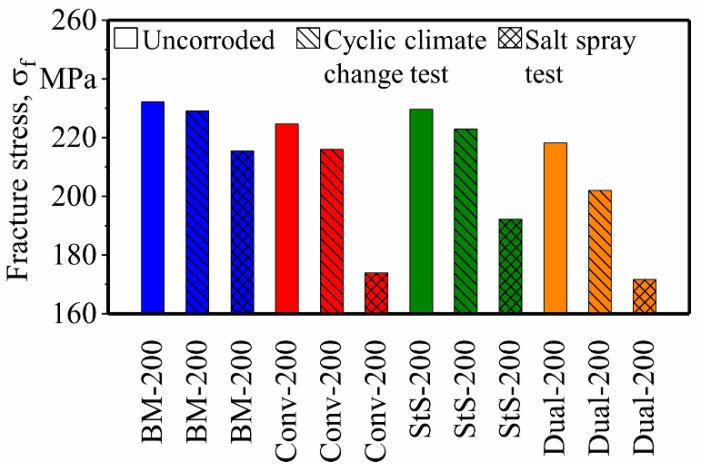
Summary of the effect of the corrosion tests on the fracture stresses of base and friction stir welded (FSW) EN AW-5754 specimens.

As shown in Figure 21, the cyclic climate change test reduced the fatigue properties of the base material, stationary shoulder, conventional and dual-rotational FSW by approximately 1%, 4%, 3% and 7%, respectively. This decrease in the fatigue properties is greater in the case of the salt spray test, which is 7%, 23%, 17% and 21% respectively for the above-mentioned specimens.

### 3.3. Fractography

**Figure 22:**
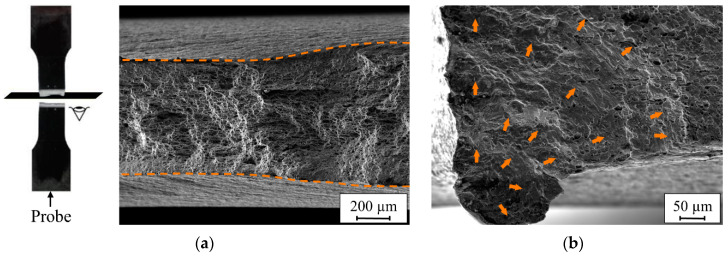
The fracture mechanisms of the finished specimens: (**a**) cyclic creep and (**b**) fatigue fracture.

The fracture surfaces of the finished FSW specimens were studied using SEM. As shown in Figure 22, the fracture mechanism of the finished FSW specimens was a combination of cyclic creep (thinning of the cross-section of the specimen is visible in Figure 22a) and fatigue fracture. In contrast to the unprocessed specimens that suffered crack initiation from the faces (weld or root), the cracks were initiated from the edges of the finished specimens (Figure 22b). This suggests that the surface finishing process was able to remove the surface defects (i.e., the potential crack initiation sites), which left the edges of the specimens as the sites with the next highest potential for crack initiation.

**Figure 23:**
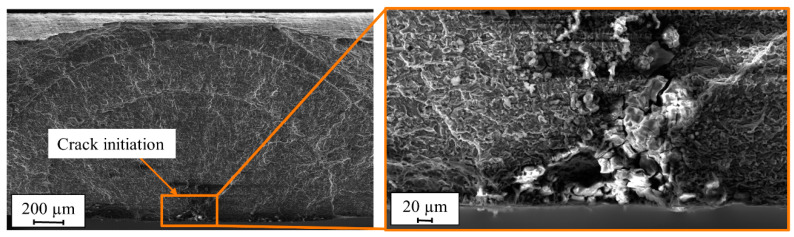
SEM images of the fractured surface of friction stir welded (FSW) EN AW-5754 specimen after the salt spray test.

**Figure 24:**
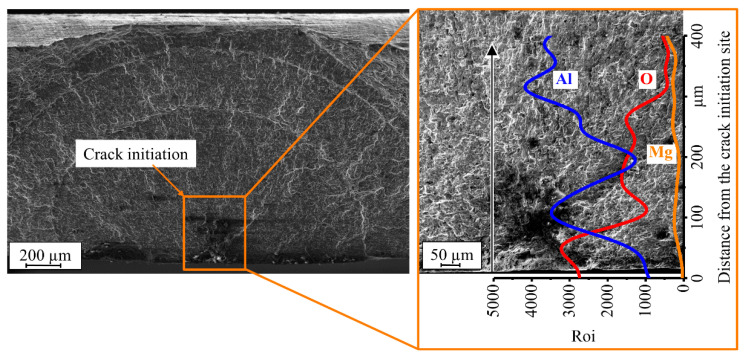
EDX analysis of the fractured surface of friction stir welded (FSW) EN AW-5754 specimen after the salt spray test.

The fractured surfaces of the precorroded specimens show that the crack was no longer initiated at the edges of the specimens like the previous finished specimens but at the corrosion pits at the surface of the specimens (Figure 23). The EDX studies were performed on the fractured surfaces of the precorroded FSW specimens using low radiation energy to better detect the oxidation on the corrosion products. The results show increasing content of oxygen (O) and at the same time decreasing content of aluminum (Al) and magnesium (Mg) from the middle of the specimens towards the corrosion pit at surface suggesting that the material was highly corroded in the pit regions (Figure 24). This reverse relationship between the oxygen and aluminum and magnesium content was observed for both corrosion tests.

### 3.4. Outlook

In the current FSW setup, it has been shown that the stationary shoulder configuration possesses superior fatigue properties compared to the conventional and dual-rotational FSW. This superiority might change if the rotational speed of the probe or tool shoulder is varied while in a parallel investigation it has been seen that the rotational speed of the probe can significantly change the fatigue properties of dual-rotational FSW specimens. Furthermore, dual-rotational configuration has the potential of significantly reducing the process forces. In the future investigations, effects of the rotational speed and direction of the probe and tool shoulder as well as process forces and temperature during the dual-rotational FSW will be investigated. In addition, further investigations on addressing the influencing factors on the fatigue properties of the FSW specimens are planned for future work.

## 4. Conclusions

In this study, effects of the surface finishing processes and corrosion on the fatigue properties of friction stir welded EN AW-5754 aluminum sheets using conventional, stationary shoulder and dual-rotational tool configurations were investigated. The fatigue results indicated a stepwise material response for all the specimens, which occurred due to the cyclic creep phenomenon. Surface of the FSW specimens with different tool configurations were milled to two different material removal depths of 200 µm and 400 µm. It was shown that the surface finishing process of 200 µm and 400 µm could increase the fracture stresses of conventional FSW from 204 to 225 and 229 MPa and dual-rotational FSW from 196 to 218 and 226 MPa, respectively. However, this increase could not be detected in stationary shoulder FSW, which showed irregular changes in the fatigue properties after the surface finishing process and fell under the base material in comparison, but it still possessed superior fatigue properties in comparison to the conventional and dual-rotational configurations with the current speed combination.

Specimens with 200 µm finished surfaces were used for salt spray and cyclic climate change tests to investigate the effect of corrosion on the fatigue behavior of FSW specimens. It was shown that the cyclic climate change test reduced the fatigue properties of the base material, conventional, stationary shoulder and dual-rotational FSW approximately 1%–7%. This decrease in the fatigue properties was even greater in the case of the salt spray test, which was 7%–21%. During both tests, the specimens suffered pitting corrosion, which was more significant in the case of salt spray test due to having longer salt spray cycles. Therefore, fatigue properties of the AW EN-5754 aluminum specimens were reduced more significantly in the salt spray test. After corrosion tests, stationary shoulder again showed superior fatigue characteristics compared to other configurations.

## Figures and Tables

**Table 1:** Chemical composition of EN AW-5754 H22.

Element	Si	Fe	Cu	Mn	Mg	Cr	Zn	Ti	Al
in %	0.4	0.4	0.1	0.5	2.6–3.6	0.3	0.2	0.15	Bal.

**Table 2:** Test parameters of the cyclic climate change test.

Step	Duration h	Temperature °C	Condition
1	24	35 ± 2	Salt spray
2	8	40 ± 2	100% relative air humidity
3	16	23 ± 2	50% ± 20% relative air humidity
4	8	40 ± 2	100% relative air humidity
5	16	23 ± 2	50% ± 20% relative air humidity
6	8	40 ± 2	100% relative air humidity
7	16	23 ± 2	50% ± 20% relative air humidity
8	8	40 ± 2	100% relative air humidity
9	16	23 ± 2	50% ± 20% relative air humidity
10	48	23 ± 2	50% ± 20% relative air humidity

**Table 3:** The weight loss measurements of the friction stir welded (FSW) EN AW-5754 specimens.

Corrosion Test	Av. Initial Weight g	Av. Final Weight g	Weight Loss %
Cyclic climate change test	25.07	25.01	0.25
Salt spray test	25.09	24.99	0.41
